# Noninvasive fluid bubble detection based on capacitive micromachined ultrasonic transducers

**DOI:** 10.1038/s41378-023-00491-6

**Published:** 2023-02-23

**Authors:** Jiawei Yuan, Zhikang Li, Qi Ma, Jie Li, Zixuan Li, Yihe Zhao, Shaohui Qin, Xuan Shi, Libo Zhao, Ping Yang, Guoxi Luo, Xiaozhang Wang, Kwok Siong Teh, Zhuangde Jiang

**Affiliations:** 1 State Key Laboratory for Manufacturing Systems Engineering, International Joint Laboratory for Micro/Nano Manufacturing and Measurement Technologies, Xi’an Jiaotong University (Yantai) Research Institute for Intelligent Sensing Technology and Systems, Xi’an Jiaotong University, 710049 Xi’an, China; 2 School of Mechanical Engineering, Xi’an Jiaotong University, 710049 Xi’an, China; 3 Shandong Laboratory of Yantai Advanced Materials and Green Manufacturing, 265503 Yantai, China; 4 School of Mechanical and Electrical Engineering, Shaanxi University of Science and Technology, Xi’an, 710049 Xi’an, China; 5 School of Engineering, San Francisco State University, San Francisco, CA 94132 USA

**Keywords:** Sensors, Electrical and electronic engineering

## Abstract

Ultrasonic fluid bubble detection is important in industrial controls, aerospace systems and clinical medicine because it can prevent fatal mechanical failures and threats to life. However, current ultrasonic technologies for bubble detection are based on conventional bulk PZT-based transducers, which suffer from large size, high power consumption and poor integration with ICs and thus are unable to implement real-time and long-term monitoring in tight physical spaces, such as in extracorporeal membrane oxygenation (ECMO) systems and dialysis machines or hydraulic systems in aircraft. This work highlights the prospect of capacitive micromachined ultrasonic transducers (CMUTs) in the aforementioned application situations based on the mechanism of received voltage variation caused by bubble-induced acoustic energy attenuation. The corresponding theories are established and well validated using finite element simulations. The fluid bubbles inside a pipe with a diameter as small as 8 mm are successfully measured using our fabricated CMUT chips with a resonant frequency of 1.1 MHz. The received voltage variation increases significantly with increasing bubble radii in the range of 0.5–2.5 mm. Further studies show that other factors, such as bubble positions, flow velocities, fluid medium types, pipe thicknesses and diameters, have negligible effects on fluid bubble measurement, demonstrating the feasibility and robustness of the CMUT-based ultrasonic bubble detection technique.

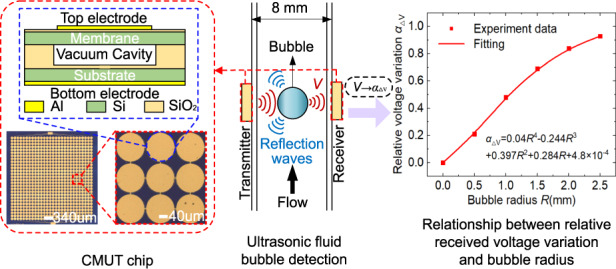

## Introduction

The ability to measure fluid bubbles in pipes is in high demand in industrial controls, pharmaceuticals, chemical production and clinical medicine because it can be leveraged to monitor the presence of air bubbles, fluid flow stability and chemical reaction processes and in some cases prevent fatal mechanical failure and life-threatening conditions^1–3^. For instance, in clinical medicine, the existence of air bubbles in extracorporeal blood circulation (ECBC) and extracorporeal membrane oxygenation (ECMO) systems can lead to air embolisms (blocking blood from passing through) when bubbles infiltrate a vein or artery, resulting in serious injuries such as strokes and heart attacks or even death^4,5^. In aerospace applications, air bubbles in propellant can cause a rapid increase in air pressure in spacecraft tanks and thus an explosion^6,7^. To date, a variety of technologies have been developed for fluid bubble detection, including image analysis^2,8^, electroresistivity^9^, optics^10^, capacitance wire-mesh sensors^11^, X-rays^12^ and ultrasound^13,14^. Compared with other methods, the ultrasonic detection approach is noninvasive, nondestructive, simple in installation and not susceptible to electromagnetic interference in complex industrial environments^15^. These unique advantages position ultrasound technology as a promising alternative for fluid bubble detection in pipes.

However, most current ultrasonic detection techniques are based on conventional bulk PZT ultrasonic transducers^16,17^, which are not suitable for applications in small pipes (e.g., diameters ≤ 10 mm)^18,19^, either for in situ, real-time or long-term monitoring, because of their shortcomings of large volume, high power consumption and difficulty in integration with ICs^20–22^. In situ, real-time and long-term fluid bubble monitoring in small pipes is extremely important in engineering situations that have tight physical spaces and require the precise and continuous monitoring of air bubbles, such as ECBC, ECMO and dialysis machines in clinical medicine or hydraulic systems in aircraft^4–7^.

Micromachined ultrasonic transducers (MUTs) provide a promising solution for the aforementioned applications because of their miniature size (their whole size could scale down to several hundred micrometers), low power consumption, low fabrication cost, and easy integration with ICs^23,24^. Currently, MUTs can be categorized into two types: capacitive and piezoelectric micromachined ultrasonic transducers (CMUTs and PMUTs)^25^. Compared to PMUTs, CMUTs take advantage of a high electromechanical coupling coefficient (~85%; 1–6% for PMUTs), wide bandwidth (~150%) and high receiving sensitivity^26–31^ (16.6 mV/Pa) and have been widely used in 3D ultrasonic imaging, ultrasonic figure printing and range finding^32,33^. Most recently, CMUTs demonstrated successful use in blood flow velocity measurement by ultrasonic Doppler techniques because of their superior performance in comparison with their conventional counterparts^34^. However, CMUT-based ultrasonic fluid bubble detection has rarely been reported thus far.

Herein, we successfully demonstrate a CMUT-based ultrasonic measurement technique for the noninvasive monitoring of fluid bubbles in small pipes. A pair of CMUTs are employed and directly clamped on two opposite sides of a pipe for ultrasound wave transmission and reception due to their miniaturized volumes. The acoustic energy attenuation and thus acoustic pressure changes induced by air bubbles are exploited to detect bubble existence and size. Closed-form expressions for the relationship between the corresponding received voltages of CMUTs and bubble sizes are established and validated using finite element simulations, which provide the theories for CMUT-based ultrasonic bubble detection. The relative received voltage variation is first proposed as the sensing response. Subsequently, bubble detection experiments in pipes with diameters less than 10 mm are implemented using our fabricated CMUT chips, and the effects of pipe diameters and thicknesses, bubble positions, flow velocities and fluid media on bubble measurement are also investigated.

## Results and discussion

### Detection principle

**Fig. 1:**
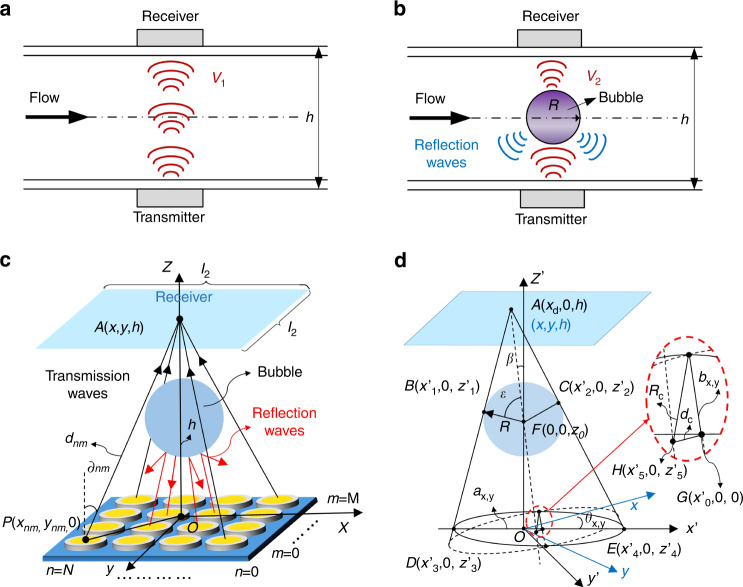
Working principle of ultrasonic fluid bubble detection for small pipes. **a** Schematic of ultrasound wave propagation without air bubbles. **b** Schematic of ultrasound wave propagation with air bubbles. **c** Principal schematic for acoustic pressure analysis. **d** Geometry schematic for analysis of the projection area of air bubbles

The sensing schematic of ultrasonic bubble detection in pipes is shown in Fig. 1. As shown in Fig. 1a, a pair of ultrasonic transducers are clamped on two opposite sides of a pipe, in which one operates as the ultrasound transmitter and the other works as the ultrasound receiver. The ultrasonic waves emitted by the transmitter pass through the pipe wall and reach the receiver, where the acoustic pressure is converted into voltage *V*_1_. As shown in Fig. 1b, compared with the situation without bubbles (Fig. 1a), the ultrasonic waves from the transmitter undergo significant reflection and energy attenuation when air bubbles are present in the propagation path, and thus the received voltage drops to *V*_2_. The received voltage variation can be utilized to realize bubble detection.

To quantitatively analyze the relationship between these variations, we derived theoretical expressions for the received voltage variation under different bubble sizes based on the theory of sound. Figure 1c shows a 3D schematic of acoustic wave propagation from a CMUT transmitter to a receiver. As the radii of CMUT cells are often less than one wavelength, each cell in the CMUT array can be assumed to be a circular piston with the same area of *s* to simplify the analysis of cell vibrations^35^. The theory of sound was used to calculate the acoustic pressure emitted by each cell, in which the acoustic waves were assumed to be continuous, and the heat loss of acoustic energy was neglected^36^. When there is no bubble in the propagation path, the acoustic pressure at point *A* (*x*, *y*, *h*) can be calculated as^37^

1 $$p_{A1}\left( {x,y,h} \right) = \mathop {\sum }\limits_{n = 1}^N \mathop {\sum }\limits_{m = 1}^M \frac{{jt_{p1}t_{p2}\rho \omega s}}{{2\pi d_{nm}}}v_{nm}e^{\left( { - jkd_{nm}} \right)} \cdot D\left( {\partial _{nm}} \right)$$

where *j* is the imaginary unit, *ρ* is the density of the fluid, *ω* is the angular frequency of acoustic waves, *k* is the wavenumber, (*n*, *m*) is the index of a CMUT cell located in row number *n* and column number *m*, *v*_nm_ is the vibration speed of a CMUT cell, and *d*_nm_ and ∂_nm_ represent the length of PA and the angle between PA and *Z*-axis, respectively, which can be given as

2 $$d_{nm} = \sqrt {\left( {x - x_{nm}} \right)^2 + \left( {y - y_{nm}} \right)^2 + h^2}$$

3 $$\partial _{nm} = \tan ^{ - 1}\frac{h}{{\sqrt {\left( {y - y_{nm}} \right)^2 + \left( {x - x_{nm}} \right)^2} }}$$

where *h* is the distance between the transmitter and receiver. *D* (∂_nm_) is the directivity function of the CMUT cell at point *P* (*x*_nm_, *y*_nm_, 0), which can be written as^37^

4 $$D\left( {\partial _{nm}} \right) = \frac{{2J_1\left( {ka\sin \partial _{nm}} \right)}}{{ka\sin \partial _{nm}}}$$

where *a* is the radius of the cells and *J*_1_ (*ka*sin∂_nm_) is the Bessel function of the first kind of the first order.

As shown in Fig. 7 in Appendix A, *t*_p1_ and *t*_p2_ in (1) are sound transmission coefficients when acoustic waves pass through the front and rear walls of pipes, respectively, which can be respectively expressed as^36^

5 $$t_{p1} = \frac{{p_2}}{{p_1}} = \frac{{4Z_1Z_3}}{{\left( {Z_1 + Z_3} \right)^2\cos ^2k_2h_2 + \left( {Z_2 + Z_3Z_1/Z_2} \right)^2{{{\mathrm{sin}}}}^2k_2h_2}}$$

6 $$t_{p2} = \frac{{p_4}}{{p_3}} = \frac{{4Z_5Z_3}}{{\left( {Z_5 + Z_3} \right)^2\cos ^2k_4h_4 + \left( {Z_4 + Z_3Z_5/Z_4} \right)^2{{{\mathrm{sin}}}}^2k_4h_4}}$$

where *p*_*1*_ and *p*_*2*_ are the incident and outgoing acoustic pressure of the front pipe wall, respectively; *p*_*3*_ and *p*_*4*_ are the incident and outgoing acoustic pressure of the rear pipe wall, respectively; *Z*_*1*_ and *Z*_*5*_ are the matching layer impedance, *Z*_*2*_ and *Z*_*4*_ are the pipe wall impedance, *Z*_*3*_ is the fluid impedance, *h*_*2*_ and *h*_*4*_ are the pipe wall thickness, and *k*_*2*_ and *k*_*4*_ are the wavenumbers of acoustic waves in the pipe wall.

Furthermore, the received voltage without bubbles in pipes can be given by multiplying the received acoustic pressure and the receiving sensitivity, *S*_c_, of CMUTs as

7 $$\begin{array}{*{20}{l}} {V_1 = \frac{{S_c}}{{l_1l_2}}\mathop {\iint}\limits_D {p_{A1}} \left( {x,y,h} \right){\rm{d}}x{\rm{d}}y} \hfill \\ {\left( {D: - l_1/2 \le x \le l_1/2, - l_2/2 \le y \le l_2/2} \right)} \hfill \end{array}$$

where *l*_1_ and *l*_2_ are the side lengths of the receiver.

When air bubbles exist in the propagation path, the incident acoustic waves are reflected at the air bubble and fluid interface due to their significant acoustic impedance difference^38^. The acoustic energy passing through the bubble can be neglected because almost all the acoustic energy incident is reflected^20^. As shown in Fig. 1d, under the aforementioned conditions, the acoustic energy transmitted by CMUT cells in the projection area of air bubbles cannot reach the receiver. To calculate the acoustic pressure at point *A* (*x*, *y*, *h*) in the presence of air bubbles, we first derive the projection area based on the geometry given in Fig. 1d. Assuming the bubble center is located on the axis of the transmitter and the bubble velocity is zero, the theoretical equation for the projection area can be written as

8 $$\frac{{\left( {x^\prime - x_0} \right)^2}}{{a_{x,y}^2}} + \frac{{y^{\prime 2}}}{{b_{x,y}^2}} = 1$$

9 $$\left[ {x^\prime ,y^\prime } \right] = \left[ {x,y} \right]\left[ {\begin{array}{*{20}{c}} {\cos \theta _{x,y}} & { - \sin \theta _{x,y}} \\ {\sin \theta _{x,y}} & {\cos \theta _{x,y}} \end{array}} \right]$$

where *x*_0_, *a*_x,y_, *b*_x,y_, *θ*_x,y_ can be given by (B.1)–(B.17) in Appendix B.

Therefore, the acoustic pressure at point *A* (*x*, *y*, *h*) with the existence of air bubbles can be obtained by summing the acoustic pressure generated by CMUT cells outside the projection area, which can be given as

10 $$\begin{array}{*{20}{c}} {p_{A2}\left( {x,y,h} \right) = \mathop {\sum }\limits_{n = 1}^N \mathop {\sum }\limits_{m = 1}^M \frac{{jt_{p1}t_{p2}\rho \omega s}}{{2\pi d_{nm}}}v_{nm}e^{\left( { - jkd_{nm}} \right)} \cdot D\left( {\partial _{nm}} \right)} \\ {\left( {\frac{{\left( {x_{nm}^\prime - x_0} \right)^2}}{{a_{x,y}^2}} + \frac{{y\prime _{nm}^2}}{{b_{x,y}^2}} \ge 1} \right)} \end{array}$$

11 $$\left[ {x_{nm}^\prime ,y_{nm}^\prime } \right] = \left[ {x_{nm},y_{nm}} \right]\left[ {\begin{array}{*{20}{c}} {\cos \theta _{x,y}} & { - \sin \theta _{x,y}} \\ {\sin \theta _{x,y}} & {\cos \theta _{x,y}} \end{array}} \right]$$

Furthermore, the received voltage in the presence of air bubbles can be written as

12 $$\begin{array}{*{20}{c}} {V_2 = \frac{{S_c}}{{l_1l_2}}\mathop {\iint}\limits_D {p_{A2}} \left( {x,y,h} \right){\rm{d}}x{\rm{d}}y} \\ {\left( {D: - l_1/2 \le x \le l_1/2, - l_2/2 \le y \le l_2/2} \right)} \end{array}$$

To avoid the influence of other factors, such as pipe diameters, pipe thicknesses, and fluid media, on the results of fluid bubble measurement, the relative voltage variation is proposed as the sensing response of the CMUTs, which is written as

13 $$\begin{array}{*{20}{c}} {\alpha _{\Delta V} = \left( {V_1 - V_2} \right)/V_1 = \frac{{\left( {\begin{array}{*{20}{c}} {\mathop {\iint}\nolimits_D {\mathop {\sum }\nolimits_{n = 1}^N \mathop {\sum }\nolimits_{m = 1}^M \frac{1}{{d_{nm}}}v_{nm}e^{\left( { - jkd_{nm}} \right)} \cdot D\left( {\partial _{nm}} \right){\rm{d}}x{\rm{d}}y} } \\ {\left( {\frac{{\left( {x_{nm}^\prime - x_0} \right)^2}}{{a_{x,y}^2}} + \frac{{y\prime _{nm}^2}}{{b_{x,y}^2}} \le 1} \right)} \end{array}} \right)}}{{\mathop {\iint}\nolimits_D {\mathop {\sum }\nolimits_{n = 1}^N \mathop {\sum }\nolimits_{m = 1}^M \frac{1}{{d_{nm}}}v_{nm}e^{\left( { - jkd_{nm}} \right)} \cdot D\left( {\partial _{nm}} \right){\rm{d}}x{\rm{d}}y} }}} \\ {\left( {\left( {D: - l_1/2 \le x \le l_1/2, - l_2/2 \le y \le l_2/2} \right)} \right)} \end{array}$$

The advantage of the sensing response definition for air bubble detection given in (13) will be further validated by experimental testings.

### Verification of the working principle

**Fig. 2:**
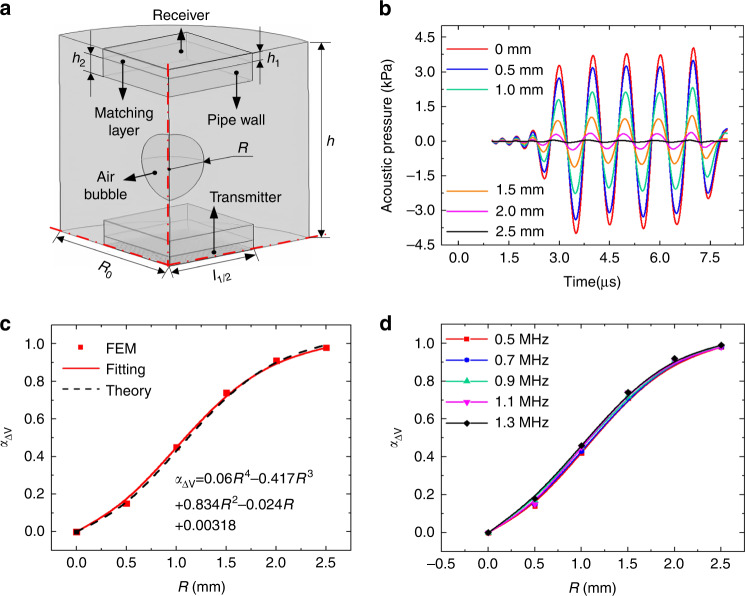
Theoretical validation of the ultrasonic measurement principle. **a** FEM model. **b** Waveforms of the received signals under bubbles with radii varying from zero to 2.5 mm. **c** Comparison between FEM simulation and theoretical analyses. **d** Simulated received voltage variations under different frequencies

To prove the feasibility of the aforementioned bubble measurement principle, we established a finite element model (FEM) to simulate acoustic pressure and received voltage variations with bubble sizes using commercially available COMSOL Multiphysics software. Figure 2a shows a quarter schematic of the FEM model, in which the pipe wall, fluid, air bubble and impedance matching layer were modeled using pressure–acoustic elements. The materials of the fluid and impedance matching layer were assumed to be silicone oil and PDMS (polydimethylsiloxane), respectively. A CMUT array with the same size as our fabricated array, 28 × 28 circular cells, was used as the ultrasound wave transmitter. The plane wave radiation boundary condition was applied to the cylindrical fluid surface, and the symmetry conditions were applied to the symmetry plane. Transient acoustic analyses were performed to obtain the receiving voltage under different sizes of air bubbles. The parameters used for the FEM simulation are shown in Table 1.

**Table 1:** The parameters used for FEM simulations

Parameters	Value
Distance between the transmitter and receiver *h*/mm	15
Size of the transmitter and receiver *l*_*1*_/mm	4.7
Thickness of matching layer *h*_*1*_/mm	1
Thickness of pipe wall *h*_*2*_ /mm	1.5
Radius of fluid domain *R*_0_/mm	3.5
Radius of cells *a*/μm	70
Impedance of matching layer *Z*_*1*_/MRayls	1.4
Impedance of pipe wall *Z*_*2*_/MRayls	3.2
Impedance of fluid *Z*_*3*_/MRayls	1.4
Receiving sensitivity *S*_c_/μV/Pa	0.1

Based on the FEM model, we first investigated the variation in acoustic pressure with bubble size. Figure 2b shows the simulated acoustic pressure changes with bubble radii varying from 0.5 mm to 2.5 mm. The received wave acoustic pressure decreased significantly with increasing bubble radii, indicating that an increase in the bubble size could cause increased ultrasound wave reflection and energy attenuation. The corresponding received voltage variation, *α*_△V_, is presented in Fig. 2c and compared with the theoretical results calculated by (13). Both the simulated and analytical results showed that the relative voltage variation, *α*_△V_, increased with bubble radii, demonstrating the correctness of our proposed theories for ultrasonic fluid bubble measurement. The relationship between them could be well fitted by a quartic polynomial function, in which the slope of the fitting curve, i.e., the relative voltage variation rate, decreased with increasing bubble radii. This is attributed to most of the acoustic energy emitted by a transducer being concentrated in the region near its axis direction. When the bubble size is larger than the central energy area, the imposed energy attenuation amplitude decreases. Furthermore, we explored the effect of frequencies on the received voltage variation, *α*_△V_, by varying it from 0.7 MHz to 1.3 MHz and keeping other parameters fixed. As shown in Fig. 2d, the results of *α*_△V_ under different frequencies almost overlapped with each other, indicating that *α*_△V_ could hardly be affected by the working frequency of the transducers. This is because the response of ultrasonic receivers is defined by the relative voltage changes, as given in (13). The frequency of ultrasound waves does affect the acoustic energy attenuation and the amplitude of the received voltage; however, it does not affect the relative voltage changes.

### Ultrasonic fluid bubble detection

**Fig. 3:**
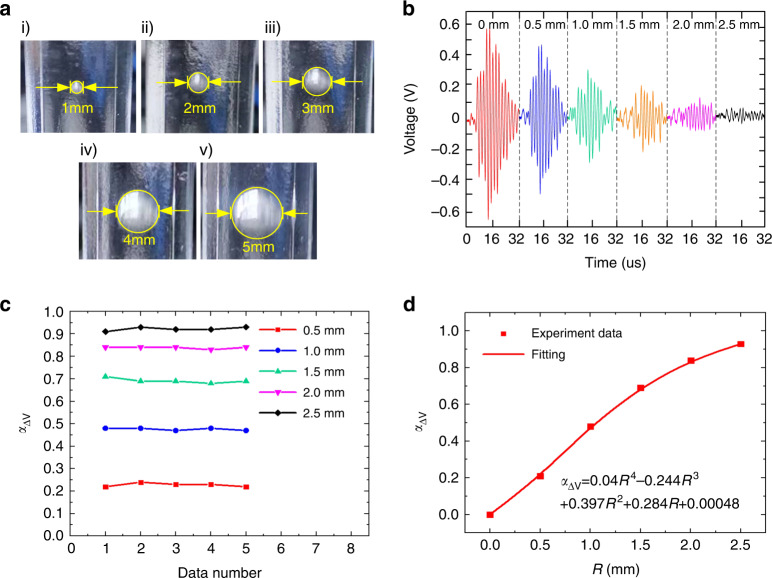
Experimental results of CMUT-based ultrasonic bubble measurement. **a** Digital images of air bubbles in small PMMA pipes with radii varying from 0.5 mm to 2.5 mm. **b** Waveforms of the received signals of CMUT chips under different sizes of air bubbles. **c** The experimental results of five repeated tests of air bubbles with different sizes. **d** Relative received voltage variation under different sizes of air bubbles

To demonstrate the feasibility of ultrasonic fluid bubble measurement in small pipes using CMUTs, we implemented experimental testing under different bubble sizes with the fluid velocity and bubble position in pipes fixed. Air bubbles of five different sizes were generated using vent holes with different radii, and their positions were kept in the center of the pipes. The radii of the generated air bubbles varied from 0.5 mm to 2.5 mm with a step of 0.5 mm, and their digital photos are shown in Fig. 3a, which were obtained using a high-speed camera. The pipe was made of PMMA, which had a thickness of 1.5 mm and a diameter of 8 mm. The fluid inside the pipe was silicone oil (50 CS), and the environmental temperature was kept at 26 °C. The testing was conducted under a 20 V DC bias and 5 V AC voltages, with an excitation frequency of 1.1 MHz and 5 pulses. The period of ultrasound emission was set to 1 ms to avoid missing air bubbles with high flow velocity. As the velocity of bubbles was less than 0.2 m/s in the experiment, the moving distance of air bubbles in pipes between the two emitted acoustic pulses was less than 0.2 mm, which was definitely covered by the ultrasound detection area of the CMUT chips (with a size of 4.7 × 4.7 mm). Therefore, the emitted acoustic pulses with 5 cycles could effectively capture the bubble and realize the detection function. For air bubble detection with increased flow velocities, the time interval between every two acoustic waves can be further shortened to ensure bubble detection accuracy, or continuous ultrasonic emission can even be adopted.

The experimental results of fluid bubble testing are shown in Fig. 3b–d. When bubbles passed through the detection field, the transmitter emitted acoustic waves continuously. When bubbles were in the center of the detection field, the reflection effect of the bubble on the acoustic wave was the strongest, resulting in the lowest received voltage signals. Figure 3b shows the received signals under different sizes of air bubbles when bubbles were in the center of the detection field. The voltage amplitude reached its maximum value when there were no air bubbles in the fluid, which decreased significantly when air bubbles appeared. This demonstrated that the presence of air bubbles in the fluid caused acoustic wave reflection and energy attenuation, which was consistent with the aforementioned theoretical analysis. Figure 3c shows the experimental results of repeated bubble testing. The received voltages had tiny fluctuations during the five times of repeated testing. The mean square errors of the received voltages with bubble radii varying from 0.5 mm to 2.5 mm were 0.0075 V, 0.0049 V, 0.0098 V, 0.0057 V and 0.0126 V, respectively, all of which were less than 5% of the average received voltage, indicating excellent repeatability. Figure 3d shows the variation in the received voltage with air bubble size. To reduce the effect of random errors in testing, the average value of five peak voltages in the received signal waveforms (as shown in Fig. 3b) was used as the received voltage. As shown in Fig. 3d, the relative voltage variation, *α*_△V_, increased with increasing bubble size, and the experimental data of *α*_△V_ were well fitted with a quartic polynomial function. Since the size of the CMUT chip was 4.7 × 4.7 mm, the sectional area of bubbles with a radius of 2.5 mm was almost the same as the area of the chip. Therefore, the acoustic wave emitted by the transmitter was almost completely reflected by the bubble with a radius of 2.5 mm, resulting in a received voltage *V*_2_ close to zero and *α*_△V_ close to 1. These experimental results agreed well with those from the FEM simulation and theoretical analysis given in Fig. 2c, validating the feasibility and efficiency of CMUT-based ultrasonic fluid bubble detection. In addition, during bubble testing, the transmitted and received CMUTs need to be aligned. The misalignment between the transmitter and receiver could lead to a reduction in the relative voltage, *α*_△V_, and thus decrease the sensing sensitivity.

### Effects of bubble positions

**Fig. 4:**
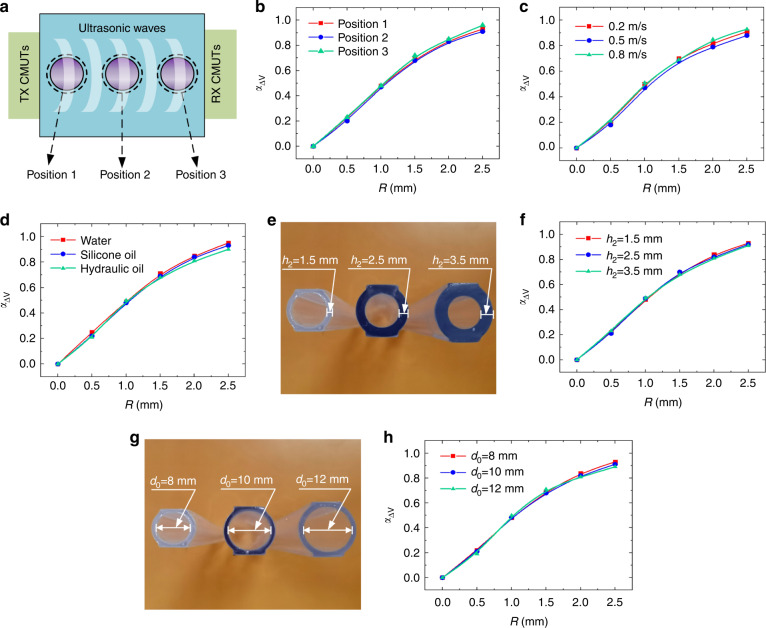
Experimental results of CMUT-based ultrasonic bubble measurement under different conditions. **a** Digital pictures of air bubbles at different positions of the pipe. **b** Received voltage variations under air bubbles with different positions. **c** Received voltage variations with bubble sizes under different flow velocities. **d** Experimental results under different flow media. **e** Digital images of different thicknesses of pipe walls. **f** Experimental measurements under different pipe thicknesses. **g** Digital images of different diameters of pipes. **h** Received voltage variations under different pipe diameters

In the experimental testing above, the position of air bubbles was controlled in the center section of the pipes. In a more realistic situation, the position of air bubbles in pipes is random. To investigate the effect of position on bubble detection, we conducted fluid bubble testing under different positions. The position of the air bubble can be controlled by adjusting the position of the vent hole, and a high-speed camera was used to confirm the position of the bubble. As shown in Fig. 4a, bubbles at three different positions were tested, that is, position 1 close to the TX CMUTs, position 2 in the center of the pipe, and position 3 far away from the TX CMUTs. Figure 4b shows the measured results under the three positions with bubble radii changing from 0.5 mm to 2.5 mm. The voltage–bubble radius curves under positions 1, 2 and 3 almost overlap with each other. This indicated that the position of air bubbles in pipes had a negligible effect on the sensing response of CMUTs. This is because, for a certain condition, the acoustic energy attenuation mainly depends on the distance of the ultrasound propagation path and bubble sizes, not the bubble position.

### Effects of flow velocities

Generally, air bubbles flow with the fluid inside pipes. Therefore, the effect of the flow velocity on ultrasonic bubble measurement was further investigated. For this, the fluid flow velocity was set to 0.2 m/s, 0.5 m/s and 0.8 m/s. The received voltages under these flow velocities with bubble radii varying from 0.5 mm to 2.5 mm were measured, the results of which are shown in Fig. 4c. For the same size of air bubbles, the voltage responses of the RX CMUTs under different flow velocities were almost the same. This illustrated that the effect of the flow velocity on the ultrasonic bubble sensing performance was negligible. This is due to the speed of acoustic waves being much greater than that of air bubbles in fluid, and the flowing bubbles can be considered static relative to the ultrasound waves during testing.

### Effects of fluid medium types

The fluid media in pipelines are different in various practical applications. To investigate the dependence of bubble sensing on fluid media, we conducted bubble testing under different fluid media, such as water, silicone oil and hydraulic oil. Figure 4d shows the measured results of the receiving voltage variation under the three types of fluid media with bubble radii varying from 0.5 mm to 2.5 mm. The received voltage variation, *α*_△V_, was almost the same for the three fluid medium types. This could be ascribed to the use of the relative voltage variation-based response definition given in (13), which largely removed the influence of fluid media. The different types of fluid media could affect the acoustic energy attenuation and the amplitude of the received voltage; however, their effect on the relative voltage changes, *α*_△V_, was negligible. The slight difference in *α*_△V_ under larger bubble sizes in Fig. 4d could be caused by the increasing noise signal-induced random error in the *α*_△V_ calculation, where the size of air bubbles approximated the CMUT chips, leading to a significant decrease in the received signal amplitude and a remarkable increase in the noise signal. These results demonstrated the robustness and advantages of our proposed CMUT-based air bubble detection method in different fluid media. Apart from the aforementioned homogeneous fluids, the proposed sensing method could also be applicable to heterogeneous fluids, such as for air bubble detection in blood in ECMO systems. This is because although heterogeneous blood induces scattering effects, resulting in increasing ultrasonic energy attenuation and reduction in the absolute received voltages, the sensing principle, which only depends on the received voltage difference between with and without the presence of air bubbles, can effectively eliminate the influence from scattering effects for a certain fluid medium.

### Effects of pipe thicknesses and diameters

Furthermore, we investigated the effect of pipe thicknesses and diameters on ultrasonic fluid bubble detection. The experiment was conducted by varying one parameter while keeping the other parameters fixed. The results of the measured voltage variation under different pipe diameters (pipe wall thicknesses) are shown in Fig. 4e–h. Figure 4e, f show the results of the voltage response under different pipe thicknesses, i.e., 1.5 mm, 2.5 mm and 3.5 mm. Figure 4g, h show the results of the measured voltage variations under different diameters of pipes, i.e., 8 mm, 10 mm and 12 mm. The experimental results of the received voltage variations under different pipe diameters (pipe wall thicknesses) almost overlapped with each other when the bubble sizes varied from 0.5 mm to 2.5 mm. These results demonstrated that both pipe diameters and pipe wall thicknesses had negligible effects on the sensing performance. This is because the increase in pipe thicknesses and diameters will mainly increase the distance between the transmitter and receiver, which decreases the absolute voltage received. However, the sensing response is defined by the relative voltage variation as given in (13), which can eliminate the influence of the absolute voltage amplitude change caused by the pipe diameters and pipe wall thicknesses.

## Materials and methods

### Design and characterization of CMUT chips

**Fig. 5:**
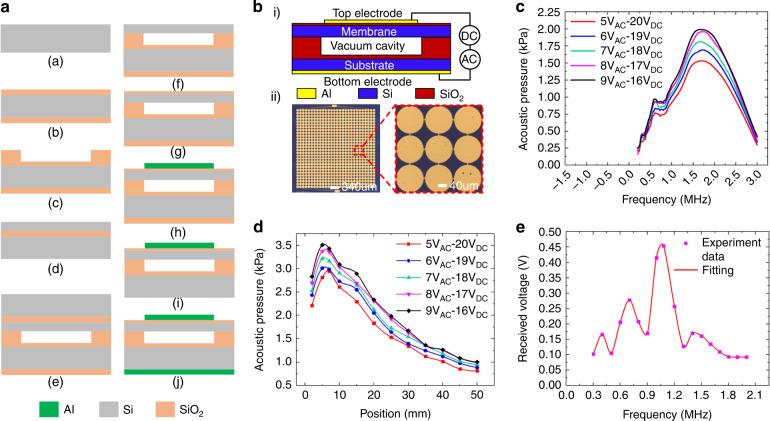
Characterization of our fabricated CMUT chips. **a** Main fabrication process of the CMUTs. **b** Structural schematic of the CMUTs. i) Cross section of a CMUT cell. ii) Optical microscope image of the CMUT array. **c** CMUT bandwidth measured at a distance of 15 mm. **d** Transmitting acoustic pressure of our CMUT chip on its axis. **e** The received voltage variation of the CMUT chip with varying frequencies

To implement fluid bubble measurements, the basic performances of the used CMUT chips were first characterized, the structural schematic of which is shown in Fig. 5b. The CMUT cell consisted of an Al top electrode, silicon membrane, SiO_2_ insulation layer, silicon substrate and Al bottom electrode (Fig. 5b–i). To meet the application of bubble detection, CMUTs were designed with low bias voltage and high natural frequency. In consideration of the device performance and fabrication process, the membrane was designed with a diameter of 140 μm and thickness of 2 μm, and the cavity was designed as 0.5 μm, which made the bias voltage as low as 40 V and the natural frequency reach 1.85 MHz. The Al top electrode had a diameter of 140 μm and a thickness of 0.4 μm to cover the vibrating area of the silicon membrane. To avoid a short circuit of the top and bottom electrodes, the SiO_2_ insulation layer under the top electrode and upon the bottom electrode with high dielectric constant had thicknesses of 0.1 μm and 0.2 μm, respectively. A silicon substrate with a resistivity less than 0.002 Ω·cm and a thickness of 725 μm was used to conduct the bottom electrode, and Al on the back of a low-resistivity silicon substrate with a thickness of 0.7 μm was used as the bottom electrode to bond with the printed circuit board. Furthermore, on the premise of ensuring the Si-SiO_2_ bonding quality, the cell pitch (distance between CMUTs cells) was as low as 148 μm to maximize the filling density and improve CMUT performance, and the size of CMUTs was designed as 4.7 × 4.7 mm to meet the application of bubble detection in pipes with an inner diameter less than 10 mm. The aforementioned CMUT-compatible CMOS was successfully fabricated using the low-temperature direct wafer-bonding technique at 350 °C^39,40^, as shown in Fig. 5a. A low resistivity silicon wafer was selected as the substrate (step a). The SiO_2_ insulation layer was generated on the low-resistivity silicon substrate by the thermal oxidation technique (step b). The SiO_2_ insulation layer was then etched to form the cavity through dry etching (step c). A silicon layer of the SOI wafer was used to form the membrane of the CMUT (step d). After cleaning, the silicon substrate and SOI wafer were bonded by the low-temperature direct wafer bonding process at 350 °C (step e). Then, mechanical polishing and dry etching technology were used to reduce the thickness of the SOI wafer to 2 μm to form the membrane (step f). A SiO_2_ layer was then deposited on the silicon membrane by the plasma-enhanced tetraethyl orthosilicate process (step g). After that, Al was sputtered and etched on the SiO_2_ insulation layer to form the top electrode (step h). Finally, hydrofluoric acid was used to remove the SiO_2_ layer at the bottom of the silicon substrate, and Al was then sputtered onto it to fabricate the bottom electrode (steps i and j).

Figure 5b-ii shows the optical microscope image of the CMUT chip, which consisted of 28 × 28 cells with a total size of 4.7 × 4.7 mm. Experimental testing using a commercially available needle hydrophone (NH2000, PA) showed that our CMUT chips had a bandwidth of 127% (−6 dB at a 15 mm distance in silicone oil (Fig. 5c). Figure 5d shows the transmitting acoustic pressure variation at the axis of the CMUT chips under different voltages. Because of the interference of the sound waves transmitted by each CMUT cell, the acoustic pressure reached a maximum at 5 mm and then decreased with increasing position^37^. The acoustic pressure increased with increasing AC voltage when keeping the total voltage amplitude of the DC and AC voltages at 25 V. This is because the membrane vibration caused by the AC voltage includes the kinetic energy and potential energy of the membrane, but the DC voltage only provides the potential energy. A maximum transmitting sensitivity of 0.6 kPa/V was obtained under a 20 V DC bias and 5 V AC voltage, which was used as the excitation voltage in the following bubble testing experiments.

Figure 5e shows the results of the received voltages of our CMUT chips under different working frequencies. To obtain an optimal voltage response for bubble detection, two CMUT chips were clamped on the opposite sides of a PMMA pipe with a diameter of 8 mm and a thickness of 1.5 mm, in which one CMUT was driven by the coaction of an AC voltage of 5 V and a bias voltage of 20 V, and the other CMUT chip was used as the receiver. Experimental testing showed that the CMUT chip had the highest voltage output at its resonant frequency of 1.1 MHz. Therefore, in the following bubble measurement, the working frequency of the CMUT chips was chosen as 1.1 MHz. The other peak voltages in the received voltage-frequency curve, such as the voltages at frequencies of 0.4 MHz, 0.7 MHz and 1.3 MHz, were caused by the crosstalk between CMUT cells due to Rayleigh–Bloch (R-B) surface waves in the fluid^41^.

### Setup for ultrasonic fluid bubble measurement

**Fig. 6:**
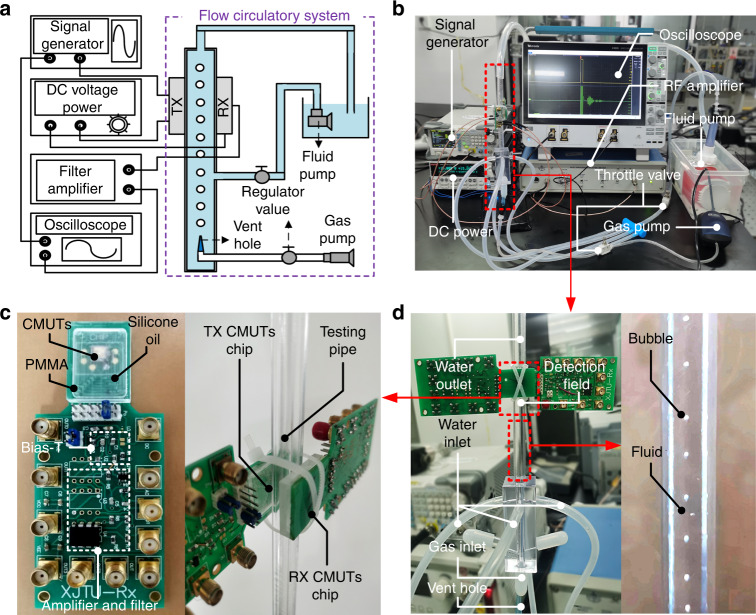
Experimental setup for ultrasonic air bubble measurement. **a** Schematic of the bubble measurement system. **b** Digital image of the experimental bubble measurement system. **c** Enlarged fluid bubble testing parts. **d** A zoomed-in picture of the fluid circulatory system

**Fig. 7:**
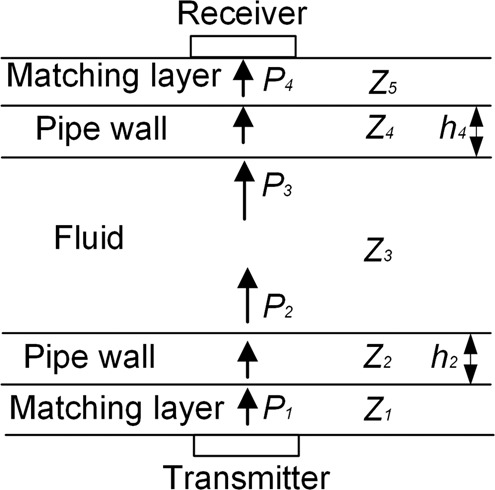
Schematic of the acoustic wave propagation process in different media

The experimental setup used for fluid bubble measurement is shown in Fig. 6. Figure 6a shows the whole system diagram, which was composed of a signal generator, a DC voltage power, an RF amplifier, an oscilloscope and a flow circulatory system. The signal generator (DG-1032Z, Rigol) and DC voltage power (2612 A, KEITHLEY) were used to provide AC and DC excitation voltages for the CMUTs, respectively. The RF amplifier (BR-640A, RIREC) was utilized for the filtering and amplification of received signals, and the oscilloscope (MSO44, Tektronix) was harnessed to display and record received signals. The fluid circulatory system was designed to produce and control fluid bubbles to be measured. Figure 6b shows a digital picture of the whole experimental setup. Figure 6d shows the enlarged fluid circulatory system. Air bubbles were injected into the pipe by the gas pump through the gas inlet. The velocity of bubble generation and the bubble sizes were controlled by the throttle valve and vent hole, respectively. The fluid was driven to flow by the fluid pump through the water inlet, and its flow velocity was controlled by the throttle valve. The fluid and air bubbles were mixed to form the measured fluid bubbles. Figure 6c shows the enlarged fluid bubble testing parts of the whole system. A pair of CMUT chips was directly clamped on the two opposite sides of the pipe for ultrasound wave transmission and reception. A PMMA cap filled with silicone oil was used as the impedance matching layer between the CMUT chips and pipes to reduce the acoustic energy attenuation at their interface. The PCB circuit board composed of a bias-T front-end and signal extraction circuits was designed to apply driving voltage and achieve a receiving signal^42^.

## Conclusion

In summary, we successfully demonstrated a noninvasive ultrasonic fluid bubble measurement technique for small pipes using CMUTs. A pair of CMUTs was used and directly clamped on two opposite sides of a pipe for ultrasound wave transmitting and receiving. The acoustic energy attenuation and thus received voltage changes caused by air bubbles were harnessed for bubble existence and size evaluation. The theoretical expressions for the received voltage and air bubble sizes were established and well validated using FEM simulations. The relative variation in the received voltages of CMUTs was proposed as the sensing response for air bubble detection. The fluid bubbles inside pipes with a diameter as small as 8 mm were measured successfully. The relative voltage variation decreased with increasing air bubble diameter. A variety of possible factors affecting fluid bubble detection, such as bubble positions, flow velocities, fluid media, pipe diameters and pipe wall thicknesses, were experimentally investigated. The results showed that all other factors had negligible effects on the sensing performance because of the sensing response definition based on relative voltage variations. This study provides a real-time, continuous and robust noninvasive fluid bubble detection technology for small pipes, which is promising in clinical medicine, industrial controls and aerospace systems.

## Appendix A

Figure 7

## Appendix B

As shown in Fig. 1(d), the XYZ coordinate system rotates along the Z-axis by an angle *θ*_*x,y*_ to obtain the X´Y´Z coordinate system. The rotation angle *θ*_*x,y*_ can be calculated as

B.1 $$\theta _{x,y} = {{\mathrm{tan}}^{-1}} \frac{y}{x}$$

The coordinate of point *A* in the X´Y´Z coordinate system is (*x*_d_, 0, *h*), where *x*_d_ can be calculated as

B.2 $$x_d = x\cos \theta _{x,y} + y\sin \theta _{x,y}$$

Then, the coordinates of point *B* (*x*´_1_, 0, *z*´_1_) and point *C* (*x*´_2_, 0, *z*´_2_) are given by

B.3 $$x_1^\prime = - R\sin \left( {\varepsilon + \beta } \right)$$

B.4 $$z_1^\prime = z_0 + R\cos \left( {\varepsilon + \beta } \right)$$

B.5 $$x_2^\prime = R\sin \left( {\varepsilon - \beta } \right)$$

B.6 $$z_2^\prime = z_0 + R\cos \left( {\varepsilon - \beta } \right)$$

where *R* is the radius of the bubble; *ε* and *β* can be given as

B.7 $$\varepsilon = {{\mathrm{cos}}^{-1}} \frac{R}{{\sqrt {x_d^2 + \left( {h - z_0} \right)^2} }}$$

B.8 $$\beta = {{\mathrm{tan}}^{-1}} \frac{{x_d}}{{h - z_0}}$$

Then, the coordinates of point *D* (*x*´_3_, 0, *z*´_3_) and point *E* (*x*´_4_, 0, *z*´_4_) can be given as

B.9 $$x_3^\prime = x_d - \frac{{h\left( {x_1^\prime - x_d} \right)}}{{\left( {z_1^\prime - h} \right)}}$$

B.10 $$x_4^\prime = x_d - \frac{{h\left( {x_2^\prime - x_d} \right)}}{{\left( {z_2^\prime - h} \right)}}$$

Therefore, the major axis and center point of the ellipse can be calculated, respectively, as

B.11 $$a_{x,y} = \left( {x_4^\prime - x_3^\prime } \right)/2$$

B.12 $$x_0^\prime = \left( {x_4^\prime + x_3^\prime } \right)/2$$

In addition, a section perpendicular to AF through point *G* was made, and point *H* (*x*´_5_, 0, *z*´_5_) is the intersection of the section and AF, which can be calculated as

B.13 $$x_5^\prime = \frac{{x_d\left( {x_1^\prime z_0 - x_2^\prime z_0 + x_0^\prime \left( {z_1^\prime - z_2^\prime } \right)} \right)}}{{ - \left( {x_1^\prime - x_2^\prime } \right)\left( {h - z_0} \right) + x_d\left( {z_1^\prime - z_2^\prime } \right)}}$$

B.14 $$z_5^\prime = - \frac{{\left[ {h \cdot x_0^\prime + \left( { - x_0^\prime + x_d} \right)z_0} \right]\left( {z_1^\prime - z_2^\prime } \right)}}{{\left( {x_1^\prime - x_2^\prime } \right)\left( {h - z_0} \right) + x_d\left( { - z_1^\prime + z_2^\prime } \right)}}$$

The minor axis of the ellipse can be expressed as

B.15 $$b_{x,y} = \sqrt {R_c^2 - d_c^2}$$

where *d*_c_ is the distance between point *G* and point *H*, which can be given as

B.16 $$d_c = \sqrt {\left( {x_5^\prime - x_0^\prime } \right)^2 + z_5^{\prime 2}}$$

*R*_c_ can be given as

B.17 $$R_c = \frac{{\left| {AF} \right|}}{{\left| {AH} \right|}}R = \frac{{R\sqrt {(x_d)^2 + (h - z_0)^2} }}{{\sqrt {(x_d - x_5^\prime )^2 + (h - z_5^\prime )^2} }}$$
